# Comprehensive Transcriptomic Analysis of the Isolated *Candida tropicalis* with Enhanced Tolerance of Furfural Inhibitor

**DOI:** 10.3390/ijms26072999

**Published:** 2025-03-25

**Authors:** Jianguang Liu, Zifu Ni, Bingyu Jiao, Yuansen Hu, Zhongke Sun, Dapeng Wu, Qipeng Yuan, Yuhuan Han, Le Wang

**Affiliations:** 1 School of Biological Engineering, Institute of Biomass Science and Engineering, Henan University of Technology, Zhengzhou 450001, China; 2 School of Environment, Henan Normal University, Xinxiang 453001, China; 3 State Key Laboratory of Chemical Resource Engineering, Beijing University of Chemical Technology, Beijing 100029, China

**Keywords:** lignocellulose, furfural, *Candida tropicalis*, tolerance, transcriptomics

## Abstract

The *Candida tropicalis* (*C. tropicalis*) named YB-3 was isolated by the Atmospheric and room temperature plasma mutagenesis from 6.5 g/L furfural tolerance. The comprehensive transcriptomic analysis of YB-3 was performed. During the stress of furfural treatment, *C. tropicalis* YB-3 protected cells from oxidative stress damage by increasing the accumulation of the glutathione reductase gene and the expression of antioxidant enzymes, with the enhancement of the inositol phosphate synthase to maintain the structural integrity and transport function of the inner membrane system, thereby affecting the cells’ tolerance. Through the gene knockout and exogenous verification, it was further confirmed that the pathways involved in the three genes of sulfate adenosine transferase gene, glutathione reductase gene, and inositol phosphate synthase gene had significant effects on improving the tolerance of the strain to furfural. The deep excavation of furfural-tolerant gene components and directional modification of *C. tropicalis* to enhance tolerance are key steps for improving the utilization rate of biomass.

## 1. Introduction

In recent years, the depletion of non-renewable energy sources such as coal and oil has spurred the emergence of low-cost lignocellulosic materials as viable alternatives, aiming to mitigate our reliance on fossil fuels. The lignocellulosic biomass presents a promising pathway for the production of second-generation biofuels and chemicals using biotechnology [1,2]. It is the best choice to replace fossil fuels [3]. Methods of converting biomass into fuel and other biomass products have been the focus of research in recent years [4,5]. Pretreatment plays a vital role in maximizing the effective utilization of biomass. However, this process also generates inhibitory compounds, such as weak acids [6], furan derivatives [7], and phenols, which have the potential to stress the activity of microorganisms involved in the subsequent processes [8,9]. Inhibitory compounds formed during the lignocellulosic pretreatments hinder biochemical processes [10,11], disrupting microbial metabolism, reducing enzyme activity, and lowering conversion efficiency, thus limiting lignocellulosic biomass’s potential as a renewable energy source [12,13]. Furfural is one of the major inhibitors produced during the pretreatment process, with a negative impact on the specific growth rate of cells [14]; it also inhibits glycolytic enzymes [15].

Furfural has been proven to decrease cell growth and ethanol productivity, and it is more sensitive to microbial growth than ethanol [16]. Furfural can reduce the content of glutathione in *Saccharomyces cerevisiae* cells, accumulate a large amount of intracellular reactive oxygen species (ROS), further damage DNA, slow down the synthesis of protein and RNA in cells, and seriously affect the normal growth and metabolism of the strain. The accumulation of ROS in cells could damage the cytoskeleton, as well as the mitochondria, vacuole membranes, and nuclear chromatin [17]. In addition, furfural can also weaken the enzyme activity of key enzymes related to the glycolysis pathway and ethanol synthesis pathway, destroy the related composition of the cell membrane, and reduce the permeability of the cell membrane, resulting in the failure of normal growth and metabolism of cells [18].

Removing inhibitors like furfural during the production process would increase production input costs. Consequently, addressing the inhibitor issue through microbial adaptation or engineering represents the most effective strategy. The inhibition of aldehydes can be alleviated by prolonging the fermentation time of *Candida tropicalis*, increasing the initial cell concentration, increasing the initial pH value, and reducing the furfural level during the evaporation of hydrolysate [19]. The integrated multi-omics investigations elucidated *Clostridium acetobutylicum*’s coordinated metabolic adaptation mechanisms to lignocellulose-derived inhibitors (furfural, formic acid, and phenol) during the butanol fermentation [20]. The yield of fumaric acid was significantly increased by the adaptive response, and the phospholipid remodeling mechanism of *Rhizopus oryzae* in response to furfural stress during the production of fumaric acid from xylose was revealed [21]. The mechanistic analysis of furfural tolerance of *C. tropicalis* identified the detoxification pathway and characterized the associated core metabolic network adaptations [22]. Using the error-prone Polymerase Chain Reaction (PCR) genome-wide recombination technology, the tolerance of *Zymomonas* mobilis with a high concentration of furfural and glucose stresses can be developed [23]. The gradient-acclimated *C. tropicalis* strains implemented a biphasic adaptation strategy, restructuring membrane fatty acid profiles to preserve membrane homeostasis while concurrently activating Adenosine Triphosphate (ATP) synthase complexes and Nicotinamide Adenine Dinucleotide Phosphate (NAD(P)H) regeneration systems through metabolic network rewiring, constituting a coordinated stress mitigation mechanism against furfural inhibition [24].

*C. tropicalis* is one of the ideal microorganisms that can grow and metabolize naturally through pretreated biomass [25,26]. Due to its rapid growth and high biomass concentration, *C. tropicalis* can use xylose as the sole carbon source with quick utilization [27,28], and it has a strong tolerance to harmful substances [29]. It showed that *C. tropicalis* has great potential for the production of biofuels and value-added chemicals in metabolic engineering, including, but not limited to, xylitol [26,30], arabitol, ethanol, dicarboxylic acid [31], citric acid, and uricase with potential applications [32,33].

In our previous studies, the selected *C. tropicalis* could use the corncob hemicellulose hydrolysate for the high-value conversion and increase the fermentation yield by optimizing the biological reaction conditions [19,34]. In this study, mutagenesis screening was employed to enhance the stress of furfural inhibitors, resulting in the acquisition of dominant strains that exhibited stable genetic tolerance to furfural. To explore the mechanism of furfural tolerance, the differential genes of the dominant strain were analyzed by transcriptome. Also, the key genes were identified to improve the tolerance. Subsequently, three specific genes of Adenosine sulfate transferase (MET3), glutathione reductase (GSH), and inositol phosphate synthase (MIPS) were chosen for knockout experiments, and comprehensive assessments of cell growth and tolerance characteristics were conducted on the gene knockout strains.

## 2. Results and Discussion

### 2.1. Enhanced Furfural Tolerance with Mutant Strains by the ARTP Mutagenesis

**Figure 1:**
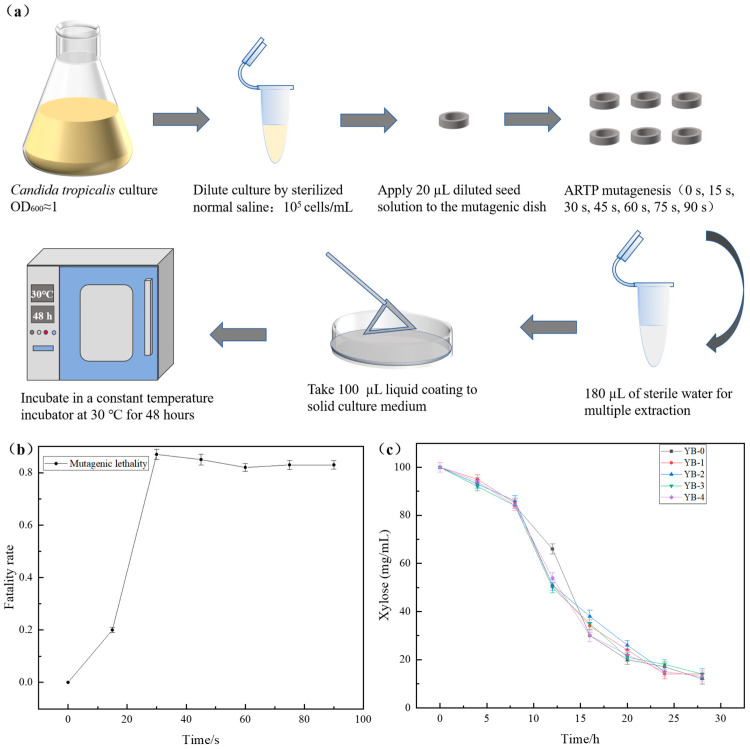
The highly furfural-tolerant strain YB-3 was screened by atmospheric and room temperature plasma (ARTP) random mutagenesis. Sterilized normal saline: sterilized 0.9% sodium chloride (NaCl) aqueous solution: (**a**) Flow diagram of ARTP mutagenesis. (**b**) Effects of different ARTP treatment times on *C. tropicalis*. (**c**) Changes in carbohydrate content of the original strain and four mutated strains in fermentation culture.

In order to analyze the genes related to furfural tolerance in *C. tropicalis*, the ARTP treatment was used to randomly mutate the *C. tropicalis* strain, aiming to screen out the strains resistant to higher concentrations of furfural (Figure 1a). As shown in Figure 1b, the lethal rate of mutagenesis reached over 85% when the mutagenesis time was 30 s. The high lethality could increase the likelihood of obtaining mutants with high yield and survival rates [35], leading to optimal mutagenesis effects. In this study, an exposure time of 30 s was deemed appropriate for achieving effective mutagenesis. Based on these results, the optimal ARTP treatment duration was determined to be 30 s. Through primary screening, 50 mutant strains with enhanced furfural tolerance were obtained. After secondary screening (Figure 1c), four strains (YB-1 to YB-4, with YB-0 as the parental strain) were selected for validation. The fermentation performance of the mutants was significantly improved, among which YB-3 exhibited the highest furfural tolerance (6.5 g/L) and was therefore selected as the optimal strain. Figure 1c demonstrates that the mutagenized strains while exhibiting enhanced tolerance to higher concentrations of furfural, maintain or even improve their fermentation performance.

### 2.2. Mutagenic Growth Fermentation and Stability Under the Furfural Stress

**Figure 2:**
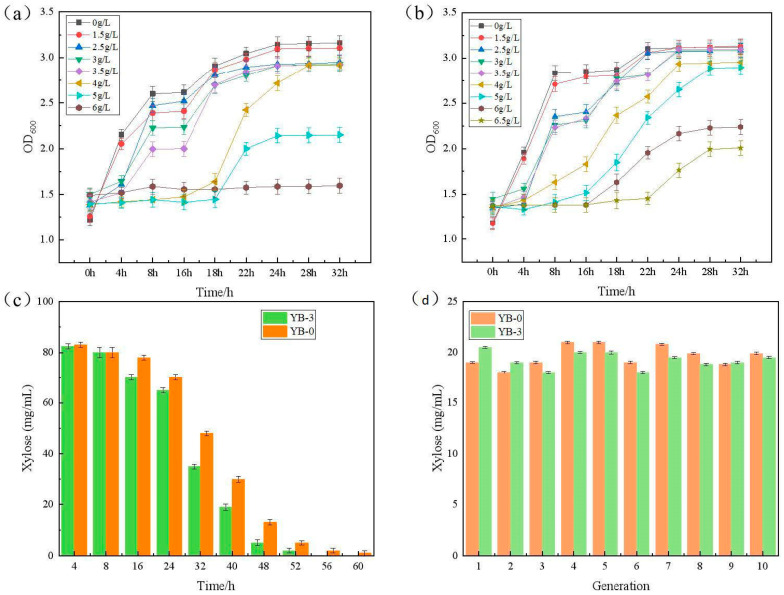
The growth curves of *C. tropicalis* strains and the stability verification of original and mutant strains: (**a**) growth curves of the original *C. tropicalis* YB-0 in different furfural concentration medium; (**b**) growth curves of the mutant strain *C. tropicalis* YB-3 in different furfural concentration medium; (**c**) comparison of the fermentation performances of xylose consumption at 3 g/L furfural concentration by the original and mutant strains; (**d**) comparison of the stability verification of xylose consumption without furfural by the original and mutant strains.

The growth curve analysis revealed critical divergence in furfural tolerance between strains: *C. tropicalis* YB-0 exhibited negligible growth at 6.0 g/L furfural, indicating complete growth inhibition at this threshold (Figure 2a). Figure 2b shows the growth curve of mutant strain *C. tropicalis* YB-3 under different concentrations of furfural stress. As illustrated in Figure 2b, *C. tropicalis* YB-3 was capable of continuing to grow even when it reached the maximum tolerance furfural concentration of 6 g/L, which was even higher than the limit for the original strain *C. tropicalis* YB-0. Furthermore, *C. tropicalis* YB-3 could grow at a furfural concentration as high as 6.5 g/L, suggesting that the mutant strain had enhanced tolerance of furfural.

The fermentation performances of *C. tropicalis* YB-3 and *C. tropicalis* YB-0 were compared under the condition of 4 g/L furfural. As shown in Figure 2c, the significantly different ability to consume xylose between the two was found from the fermentation of 16 h. The ability of *C. tropicalis* YB-0 to consume xylose was significantly lower than that of the mutant strain *C. tropicalis* YB-3. The original strain required more time to consume the same amount of xylose compared to the mutant strain. This proves that the tolerance of YB-3 to furfural is significantly enhanced after forward mutation screening.

As shown in Figure 2d, the original strain *C. tropicalis* YB-0 and the mutant strain *C. tropicalis* YB-3 were continuously fermented for 10 consecutive generations without furfural, and the fermentation performances of xylose consumption were measured after the growth to the stable stage. Following ten consecutive generations of subculture, the mutant strain maintained stable xylose consumption capacity (fluctuation amplitude < 5%), with coefficients of variation (CV) below 3% for critical fermentation parameters (productivity and yield), confirming its excellent genetic stability.

Furthermore, the fermentation performance of the mutant strain *C. tropicalis* YB-3 in xylitol production was compared at different furfural concentrations. As illustrated in Figure S1, at the furfural concentrations of 1 g/L and 2 g/L, fermentation performances of the strain improved with the increase in furfural concentration. At the furfural concentrations of 3 g/L and 4 g/L, the fermentation performances decreased as the furfural concentration increased. Therefore, a furfural concentration of 3 g/L was identified as the optimal level for investigating the strain’s tolerance.

### 2.3. Transcriptome Analysis and Identification of Furfural Tolerance-Related Genes

**Figure 3:**
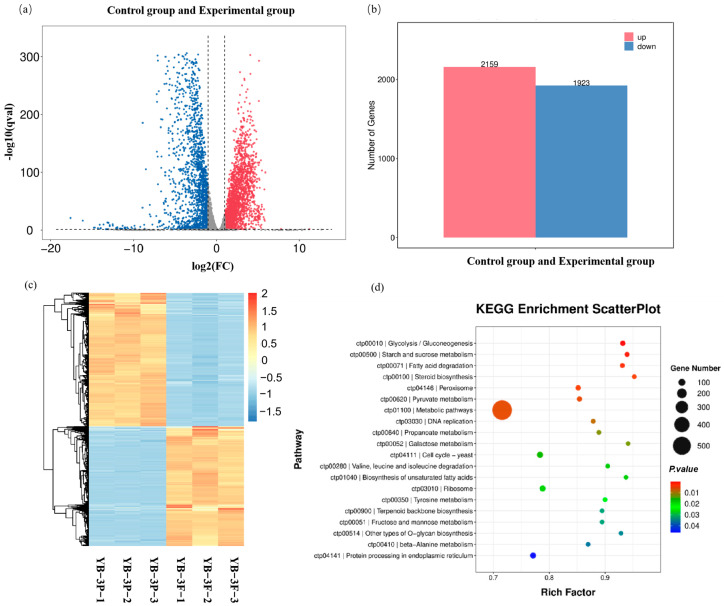
Analysis of transcriptome bioinformation and correlation of differentially expressed genes: (**a**) Volcano plot of differential genes (top 20 genes); (**b**) Up-regulated and down-regulated genes; (**c**) Heat map of hierarchical clustering analysis of differentially expressed genes. Control group: YB-3P1–P3; Experimental group: YB-3F1–F3; YB: mutagenesis treatment; 3: the strain screening number; P: blank control group (e.g., P1-P3 represent triplicate controls); F: experimental group (e.g., F1–F3 represent triplicate test samples). (**d**) Bubble plot analysis of KEGG functional enrichment of differentially expressed genes.

To investigate the genes related to the furfural tolerance in the mutant strain *C. tropicalis* YB-3, a comparative transcriptome analysis was conducted between the strains treated with and without furfural. As shown in Figure 3a,b, the volcano plot of differentially expressed genes of *C. tropicalis* YB-3 under the concentration of 3 g/L furfural showed that the distribution of differential genes was roughly symmetrical, with a higher number of up-regulated genes (2159) compared to down-regulated genes (1923).

The differentially expressed genes (DEGs) were hierarchically clustered based on their expression levels in strains with and without furfural treatment. We focused on the clustering results of DEGs with enhanced transcription in the furfural-tolerant strain *C. tropicalis*. In (Figure 3c), the hierarchical cluster analysis revealed that the DEGs exhibited enhanced expressions in the presence of furfural under both treated and untreated conditions. Notably, the furfural-treated *C. tropicalis* YB-3 showed increased expression of DEGs, which were linked to multiple up-regulated KEGG pathways related to furfural tolerance. In Figure 3d, the KEGG functional enrichment analysis showed that the pathways significantly regulated under furfural stress mainly included proteasome, protein processing in the endoplasmic reticulum, peroxisome, α-linolenic acid metabolism, biosynthesis of unsaturated fatty acids, and ubiquitin-mediated proteolysis (*p* < 0.01), indicating that these pathways were the key responses of furfural-treated strain *C. tropicalis* YB-3-N to furfural tolerance.

**Figure 4:**
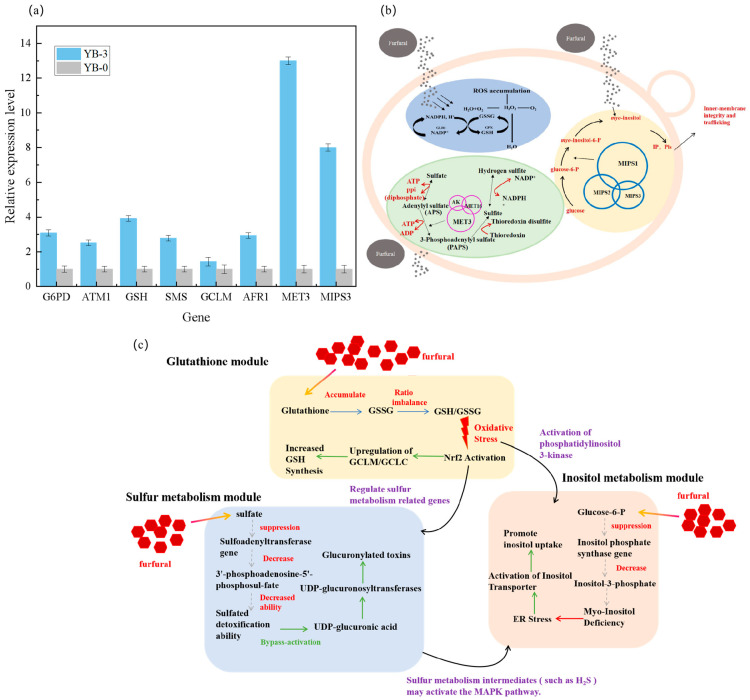
(**a**) The relative expression of related genes in YB-3 and YB-0 was verified by qPCR. (**b**) Analysis of key genes related to furfural tolerance The clearance pathway of reactive oxygen species, NADP^+^/NADPH, and the changes in differentially expressed genes in metabolic pathways. (**c**) MET3, GSH, MIPS gene tolerance signaling pathway map. The gray dashed lines indicate inhibitory effects, the red arrows represent stimulatory effects, and the green arrows denote enhancement effects.

To further validate the accuracy of transcriptome data, the expression levels of differential genes obtained through RNA-seq analysis were verified. In this study, eight differentially expressed genes and one internal reference gene from the 3.0 g/L furfural treatment were selected for fluorescence quantitative PCR detection. In Figure 4a, the fluorescence quantitative PCR data of eight differentially expressed genes were analyzed. It was found that the expression level of the differentially expressed genes was consistent with the expression trend in the transcriptome data, confirming that transcriptome sequencing results were accurate and reliable.

Furfural could cause cell damage correlated with ROS accumulation, affecting mitochondria, vacuolar membranes, the actin cytoskeleton, and nuclear chromatin [36]. When yeast cells encountered furfural, they reduced it to furfuryl alcohol, protecting themselves from its effects and repairing any damage [37]. The furfural inhibitor induced oxidative stress in *C. tropicalis* cells, a common resistance response in aerobic cells. Glutathione (GSH) is a prevalent cellular thiol crucial for many processes and protects against xenobiotics, carcinogens, and radiation stress. GSH aids defense against reactive oxygen species by supporting both non-enzymatic and enzymatic processes. It scavenged free radicals and acted as a cofactor for enzymes, such as glutathione peroxidase and reductase, in antioxidant mechanisms [38]. Glutathione reductase recirculated oxidized glutathione into a reduced form, catalyzing the conversion of glutathione disulfide (GSSG) into GSH NADPH-dependent glutathione reductase gene (CTRG _ 02682) and glutathione synthase gene (CTRG_03089). Under the furfural stress, the transcriptional levels of these two genes increased, enhancing the removal of excess reactive oxygen species to maintain cellular reduction and protect against damage (Figure 4b). Adding glutathione increased endogenous synthesis and improved strain tolerance to 3-hydroxypropionic acid, organic acids, and furfural, revealing a GSH-dependent detoxification mechanism [39]. The 6-phosphogluconate dehydrogenase gene (CTRG_03660) in the pentose phosphate pathway aided the glutathione synthesis in *C. tropicalis* YB-3. By boosting sulfur metabolism and pentose phosphate pathway flux, it ensured NADPH and ATP availability for GSSG-to-GSH conversion, maintaining redox balance and enhancing furfural tolerance. In this study, differentially expressed genes involved in arginine and proline metabolism were up-regulated in *C. tropicalis* YB-3 (Table S1).

In this study, differentially expressed genes involved in arginine and proline metabolism were up-regulated in *C. tropicalis* YB-3. Under furfural induction, the transcription levels of proline dehydrogenase (CTRG_06001), 1-pyrroline-5-carboxylic acid dehydrogenase (CTRG_06088), glutamate-5-semialdehyde dehydrogenase (CTRG_05627), and glutamate-5-kinase (CTRG_05787) of *C. tropicalis* YB-3 were up-regulated (Table S1). During osmotic stress, proline accumulates in bacterial and plant cells for protection. Yeast cells produced proline from glutamate in the cytoplasm using the same pathway as bacteria and plants, and they also converted excess proline back to glutamate in mitochondria. Proline has stress protection activity [40]. The enhanced regeneration of ATP and NADH is thought to be related to resistance to inhibitory compounds in lignocellulosic hydrolysates [41], an adaptive rapid response to NADPH consumption under stress conditions. Under furfural stress, the transcription levels of sulfate adenosine transferase gene (CTRG_03661), sulfate adenosine kinase gene (CTRG_05262), salt-tolerant protein HAL2 gene (CTRG_02963), and sulfite reductase gene (CTRG_01492, CTRG_01493) involved in sulfur metabolism pathway in *C. tropicalis* YB-3 were significantly enhanced (Table S1). The 6-phosphogluconate dehydrogenase gene (CTRG_03660) in the pentose phosphate pathway aids in GSH synthesis, suggesting that *C. tropicalis* YB-3 enhanced NADPH and ATP production for GSSG-to-GSH conversion by boosting sulfur metabolism and pentose phosphate pathway flux. This maintained the intracellular redox balance and enhanced the furfural tolerance.

The furfural treatment of *C. tropicalis* disrupted intracellular redox balance, leading to ROS accumulation. This, in turn, may cause unfolded or misfolded proteins to build up in the endoplasmic reticulum, ultimately triggering the endoplasmic reticulum stress (ERS) response. The heat shock protein family (HSPs) aided in amino acid oxidation reversal and protein folding during the unfolded protein response (UPR). When proteotoxic overload exceeded cellular degradation capacity, sustained stress-activated Jnk kinase-mediated apoptosis via proteasome up-regulation, mirroring cellular responses to DNA damage [42]. Proteasome homeostasis, regulated through Rpn4-dependent negative feedback in *Saccharomyces cerevisiae*, became compromised under furfural stress through dual mechanisms: Rpn4 degradation inhibition and proteasome impairment from either genetic mutations or ER-derived misfolded proteins, ultimately causing growth defects [43]. When furfural was used on *C. tropicalis*, genes related to unfolded/misfolded ER proteins, heat shock proteins (CTRG_01443), and chaperones (CTRG_04372) were up-regulated (Table S1).

Through the KEGG database analysis [24] (Figure 3d), the furfural-resistant strain *C. tropicalis* YB-3 was treated with furfural, oxidative stress-regulated sulfur metabolism-related genes (such as thioredoxin) through Nrf2. Sulfur metabolism intermediates may activate the MAPK pathway, and AKT activation supports cell survival and counteracts inhibitor toxicity (Figure 4c). The qPCR results are shown in Figure 4a to verify that these pathways are consistent with transcriptome findings. The transcription levels of the eight genes are shown in Table S2. Adenosine sulfate transferase, glutathione reductase, and inositol phosphate synthase were involved in sulfur metabolism, glutathione metabolism, and inositol phosphate metabolism, respectively, and synergistically maintained cell redox and metabolic homeostasis.

In the presence of furfural, *C. tropicalis* cells experienced redox imbalance, resulting in the accumulation of ROS. This ROS accumulation led to the buildup of unfolded or misfolded proteins in the endoplasmic reticulum lumen, ultimately inducing oxidative stress. Treating the tolerant strain YB-3 with 3.0 g/L furfural supplied adequate NAD(P)H and ATP, which helped maintain optimal redox balance within the cell. This treatment also enabled swift removal of misfolded proteins and preserved cell membrane integrity (Figure 4b). Three highly expressed up-regulated genes in the tolerant strain *C. tropicalis* YB-3 were screened: the adenosine sulfate transferase gene, glutathione reductase gene, and inositol phosphate synthase gene for further tolerance verification. YB-3 is a forward-mutated strain obtained through screening, and transcriptome analysis revealed that the genes exhibiting significantly elevated transcription levels are positively correlated key genes.

### 2.4. Identification of Furfural Tolerance Gene Knockout Verification

Key genes (MET3, GSH, MIPS) involved in furfural stress response were selected based on transcriptomic analysis. The homologous arm pairs GSH-T1/GSH-T2 (for the GSH gene), MIPS-T1/MIPS-T2 (for the MIPS gene), and MET3-T1/MET3-T2 (for the MET3 gene) were amplified via PCR using *C. tropicalis* genomic DNA as the template. Agarose gel electrophoresis revealed distinct bands of approximately 1000 bp (Figure S2a–c), which closely matched the theoretical size of the target fragments. Subsequently, the Kanamycin resistance gene (Kanr) was amplified from the plasmid pET28a template using primer pairs GSH-Kanr-F2/R2, MIPS-Kanr-F2/R2, and MET3-Kanr-F2/R2. A distinct band of 900 bp was observed (Figure S2a–c), corresponding to the expected size of the Kanr coding sequence.

The primers GSH-Kanr-F2 and GSH-Kanr-R2 were used to verify the kanamycin marker site-specific insertion for the Δ*GSH* mutant. The PCR product was assessed by agarose gel electrophoresis, which showed a band of 1000 bp (Figure S3a), indicating the single-knockout of GSH. The presence of the kanamycin marker site-specific insertion for the MET3 and MIPS mutants colony genome was verified with primers MET3-Kanr-F2/MET3-Kanr-R2 and MIPS-Kanr-F2/MIPS-Kanr-R2, respectively. The PCR products were, respectively, examined by agarose gel electrophoresis, which showed the band of 1000 bp (Figure S3b,c), indicating that the single-knockout of Δ*MET3* and Δ*MIPS.*

**Figure 5:**
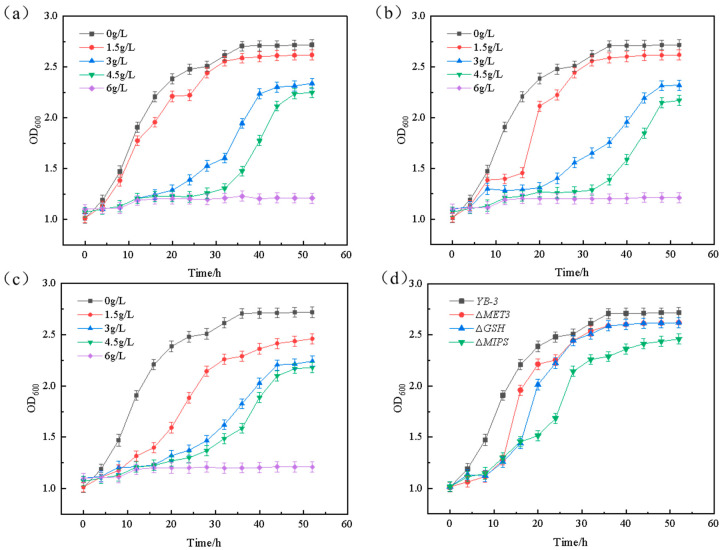
Tolerance comparison of ∆*MET3*, ∆*GSH*, and ∆*MIPS* with *C. tropicalis* YB-3: (**a**) the growth of different furfural concentrations of Δ*MET3*; (**b**) the growth of different furfural concentrations of Δ*GSH*; (**c**) the growth of different furfural concentrations of Δ*MIPS*; (**d**) the growth of Δ*MET3*, Δ*GSH*, and Δ*MIPS* and *C. tropicalis* YB-3 at 2 g/L concentration of furfural.

Knockout mutants (Δ*MET3*, Δ*GSH*, Δ*MIPS*) were constructed via homologous recombination and subsequently compared with the wild-type strain for furfural tolerance across concentration gradients (0–6 g/L) (Figure 5).

In the absence of furfural, there was almost no difference in the growth of Δ*MET3*, Δ*GSH*, and Δ*MIPS* mutants and strain YB-3 in the YPD medium without furfural stress. When the gene knockout strains were cultured under 1.5 g/L furfural stress, their growth rates were slower than that of YB-3. However, the time taken for them to reach the end of their growth phase was nearly identical to that of strain YB-3. In addition, in the presence of furfural, the growth rate of the three gene knockout strains was lower than that of strain YB-3, confirming the essential role of these genes in maintaining cellular resistance. Notably, strain YB-3 maintained superior growth performance compared to the knockout strains even under 3 g/L furfural stress, though significant growth inhibition was observed at this elevated concentration. This suggests that these three genes play a crucial and beneficial role in the strain’s tolerance to furfural.

Studies have shown that ATP and NADPH regeneration enhance resistance to inhibitory compounds in lignocellulosic hydrolysates [44]. The sulfate adenosine transferase is involved in the supply of ATP and NADPH and increases ATP and NADPH, which is an adaptive and rapid response to NADPH consumption under stressful environmental conditions [40]. It has been reported that the increase in GSH conversion and synthesis requires a large amount of ATP and NADPH to maintain the balance of the intracellular redox environment and improve tolerance [17]. Glutathione transferase genes protect cells from ROS damage in stress responses. MIPS, involved in phosphatidylinositol phosphate synthesis, maintains membrane integrity and transport, influencing cell growth regulation [45]. The pathways associated with the stress-resistant elements of the three key genes—MET3, GSH, and MIPS—played a pivotal role in the strain’s ability to resist furfural. This allowed the strain to exhibit superior growth and metabolism even in the presence of higher concentrations of furfural.

### 2.5. The Exogenous Verifications of Genes to the Furfural Tolerance

**Figure 6:**
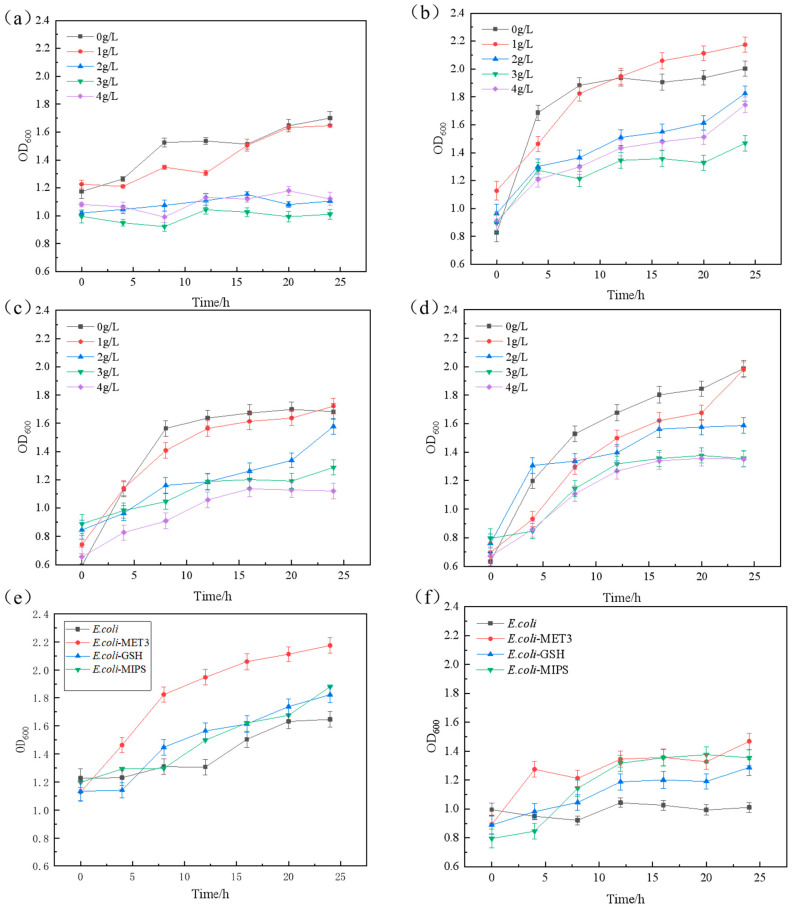
(**a**) The growth of *E. coli* at different furfural concentrations; (**b**) the growth of *E. coli*-MET3 at different furfural concentrations; (**c**) the growth of *E. coli*-GSH at different furfural concentrations; (**d**) the growth of *E. coli*-MIPS at different furfural concentrations; (**e**) the tolerance comparison of *E. coli*-MET3, *E. coli*-MIPS, and *E. coli*-GSH with *E. coli* under the concentrations of furfural at 1 g/L; (**f**) the tolerance comparison of *E. coli*-MET3, *E. coli*-MIPS, and *E. coli*-GSH with *E. coli* under the concentrations of furfural at 3 g/L.

To further verify the role of the excavated genes in furfural tolerance, we conducted heterologous expression of three key genes (MET3, GSH1, MIPS) from *C. tropicalis* in *E. coli* DH5α, generating engineered strains *E. coli*-MET3, *E. coli*-MIPS, and *E. coli*-GSH. The growth of *E. coli*, *E. coli*-MET3, *E. coli*-MIPS, and *E. coli*-GSH at different concentrations of furfural (Figure 6a–d) and the tolerance comparison of *E. coli*-MET3*, E. coli*-MIPS, and *E. coli*-GSH with *E. coli* under the concentrations of furfural at 1 g/L (Figure 6e) and 3 g/L (Figure 6f) were identified.

*E. coli*-MET3, *E. coli*-MIPS, and *E. coli*-GSH showed no significant difference in growth compared to *E. coli* in LB medium without furfural. When cultured in the presence of 1 g/L furfural inhibitor, the lag phase of the three recombinant strains was significantly shorter than that of *E. coli*, and the recombinant bacteria entered the logarithmic growth phase earlier. In LB medium without furfural, the growth of *E. coli*-MET3, *E. coli*-MIPS, and *E. coli*-GSH exhibited no significant difference compared to that of *E. coli*. However, when cultured in the presence of 1 g/L furfural, the lag phase of these three recombinant strains was significantly shorter than that of *E. coli*, allowing them to enter the logarithmic growth phase earlier. Further verifications of the effects of the three key stress-resistant elements (MET3, GSH, and MIPS) on furfural tolerance confirmed that the introduction of exogenous furfural-tolerant components could enhance the strain’s resistance to furfural.

Under the presence of 1 g/L furfural, the recombinant strains demonstrated higher final biomass compared to the wild-type strain (Figure 6e) while showing similar biomass levels at 3 g/L concentration (Figure 6f). Notably, the *E. coli*-MET3, *E. coli*-MIPS, and *E. coli*-GSH variants not only overcame the furfural-induced lag phase more rapidly than the *E. coli* but also achieved superior final growth performance and metabolic activity with increased biomass accumulation, thereby demonstrating enhanced cellular proliferation capabilities.

**Figure 7:**
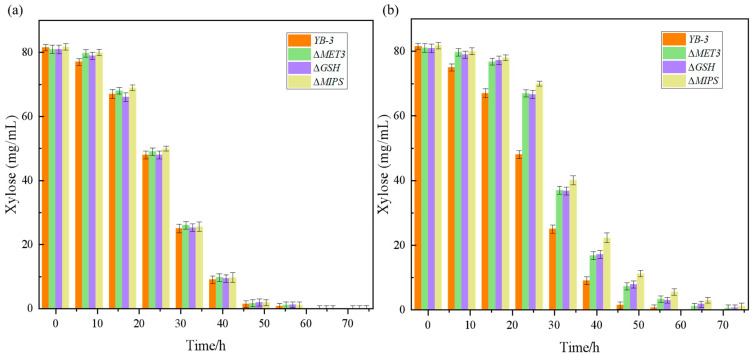
(**a**) Comparison of fermentation performance between the condition-related gene knockout strains and YB-3 strains without furfural. (**b**) Comparison of fermentation performance between the condition-related gene knockout strains and YB-3 strains on the furfural concentrations of 2 g/L.

To further validate the impact of these three key genes (MET3, GSH, and MIPS) on fermentation, the fermentation performance of the gene knockout strains and the YB-3 strain under both furfural-free and furfural-containing conditions was analyzed. As shown in Figure 7, under furfural-containing conditions, the deletion of all three related genes affected fermentation performance, with the deletion of the GSH gene having a particularly significant impact on furfural tolerance.

In summary, it knocked out three key stress-resistant genes (MET3, GSH, and MIPS) using homologous recombination, resulting in the creation of mutant strains ∆MET3, ∆GSH, and ∆MIPS, respectively. Under 2 g/L furfural stress, the cellular growth of strain YB-3 exhibited significant differences compared to its gene-knockout mutants. Under identical furfural concentrations, the knockout mutants showed marked growth inhibition, demonstrating that the YB-3 possesses superior tolerance to furfural stress. When exposed to 2 g/L furfural, all three mutant strains displayed significantly slower specific growth rates than the parental YB-3 strain. The recombinant plasmids pCaEXP-MET3, pCaEXP-MIPS, and pCaEXP-GSH were further constructed into *E. coli* DH5α to obtain strains *E. coli*-MET3, *E. coli*-MIPS, and *E. coli*-GSH, respectively. When cultured in an environment containing 1 g/L furfural inhibitor, the recombinant strain exhibited a significantly shorter lag phase compared to *E. coli*, enabling it to enter the logarithmic growth phase more rapidly and achieve a higher growth rate than the original strain. This further confirms that the three pathways (MET3, GSH, and MIPS) significantly enhance the strain’s tolerance to furfural.

## 3. Materials and Methods

### 3.1. Strains and Plasmids

The strains used in this experiment were *C. tropicalis* preserved in the laboratory. The furfural-tolerant mutant strain *C. tropicalis* YB-3 was developed through atmospheric and room temperature plasma (ARTP) mutagenesis using the ARTP mutagenesis system (Weens Technology Co., Ltd., Beijing, China) on the parental strain. Plasmid pMD20 was purchased from TaKaRa Biotechnology Co., Ltd. (Dalian, China); plasmid pET28a was purchased from Sangon Biotech (Shanghai) Co., Ltd. (Shanghai, China); plasmid pCaEXP was purchased from Wuhan Miaoling Biotechnology Co., Ltd. (Wuhan, China); and *E. coli* DH5α was purchased from Qingke Biotechnology Co., Ltd. (Chengdu, China).

The LB liquid medium contained yeast powder 5 g/L, tryptone 10 g/L, and sodium chloride 10 g/L. The YPD liquid medium contained yeast powder 10 g/L, peptone 20 g/L, and glucose 20 g/L. The LB solid medium contained yeast powder 5 g/L, tryptone 10 g/L, sodium chloride 10 g/L, and agar (solid) 20 g/L. The YPD solid medium contained yeast powder 10 g/L, peptone 20 g/L, glucose 20 g/L, and agar (solid) 20 g/L. The selection medium contained LB medium with antibiotics (50 mg/L ampicillin/kanamycin). The furfural tolerance medium contained peptone 20 g/L, yeast powder 10 g/L, glucose 20 g/L, furfural (0–6) g/L. The seed medium contained glucose 20 g/L, xylose 20 g/L, yeast powder 10 g/L, diammonium hydrogen phosphate 3 g/L, dipotassium hydrogen phosphate 5 g/L, magnesium sulfate 0.5 g/L. The fermentation medium contained xylose 110 g/L, yeast powder 10 g/L, diammonium phosphate 3 g/L, dipotassium hydrogen phosphate 5 g/L, magnesium sulfate 0.5 g/L. All of the media were sterilized at 115 °C for 30 min.

### 3.2. Mutagenesis Experiment by the ARTP

The ARTP mutagenesis was performed using *C. tropicalis* as the parental strain. Exponentially growing cells from seed culture medium were harvested, washed, and resuspended in sterile saline (0.9% sodium chloride aqueous solution) to achieve a 10^5^-fold dilution. (An amount of 9 mL of 0.9% sterile saline solution was dispensed into each test tube, followed by sterilization (115 °C for 30 min) and stored for later use. An amount of 1 mL of the exponential-phase seed culture was transferred into the first 9 mL saline tube and vortexed thoroughly for 30 s to prepare a 10-fold dilution gradient suspension. Sequentially, 1 mL of the suspension was transferred from the current dilution tube to the next 9 mL saline tube. Thorough mixing was ensured after each transfer. This process was repeated five times to achieve a final dilution of 10^5^). Aliquots (20 μL) of the diluted suspension were uniformly spread on sterilized mutagenesis plates. The multifunctional plasma mutagenesis system was operated under optimized parameters: discharge power 120 W, working pressure 0.4 Pa, and helium gas flow rate 12.0 SLM (standard liters per minute). Ten labeled mutagenesis plates (A-J) were sequentially processed. Treatment durations of 0 (control), 15, 30, 45, 60, 75, and 90 s were applied, with duplicate plates per exposure time. Post-mutagenesis, cells were eluted from each plate using 180 μL sterile phosphate-buffered saline (PBS) (pH 7.4) through three sequential wash cycles. The eluates were pooled into pre-chilled centrifuge tubes. Aliquots (100 μL) were then spread on a seed solid medium and incubated at 30 °C for 48 h. The growth status of colonies on the plate was observed, and the number of colonies was recorded. The formula for calculating the mortality rate is as follows:

$$Z=(1-\frac{X_{1}}{X_{0}})\times 100\%$$

*Z* indicates the lethality of the strain; *X*_1_ represents the number of single colonies after mutagenesis; *X*_0_ is the number of single colonies without mutagenesis under the same conditions.

The parental strain *C. tropicalis* was subjected to three parallel treatments under the optimal mutagenesis parameters. Following seed culture, the cells were plated onto a solid medium containing the maximum tolerated concentration (MTC) of furfural and incubated at 30 °C for colony morphology observation. Fifty colonies with growth advantages were selected through primary screening. After secondary screening via slant subculturing, four phenotypically stable positive mutants (designated YB-1 to YB-4) were ultimately identified. The mutants and wild-type *C. tropicalis* were synchronously inoculated into a seed medium (30 °C, 24 h) and then transferred to a fermentation medium containing furfural at 6% (*v*/*v*) inoculum size. Fermentation was conducted under controlled conditions (30 °C, 170 r/min, 36 h). Time-course samples were collected, and xylose consumption was quantified by high-performance liquid chromatography (HPLC) (Shimadzu Corporation, Kyoto, Japan) to evaluate metabolic efficiency differences relative to the wild-type strain.

After the fermentation, cells were replated on a furfural-stressed medium to verify tolerance stability. The superior mutants were subjected to 10 successive subcultures (6% inoculum size, 30 °C, 170 r/min).Longitudinal data comparisons were performed to validate the hereditary stability of metabolic traits.

For the HPLC analysis, all samples were filtered through 0.22 μm membrane filters and analyzed using a Shimadzu HPLC system. The analysis was performed on a Waters Sugar Chromatography Column (Milford, CT, USA) maintained at 80 °C with a refractive index detector (RID). The mobile phase consisted of purified water, delivered at a flow rate of 0.5 mL/min, and an injection volume of 10 µL was applied. Calibration curves were established using standard solutions of xylose and xylitol at varying concentrations.

### 3.3. The Growth Curve, Fermentation Performance, and Stability Experiments of Mutant Strains Under Furfural Stress

The single colony of the original strain *C. tropicalis* YB-0 and the mutant strain *C. tropicalis* YB-3 were inoculated in YPD liquid medium without furfural. The cells were cultured at 30 °C overnight until OD_600_ reached 1.2–1.5. Afterward, the 6% (*v*/*v*) inoculum size yeast liquid was inoculated into 50 mL of liquid YPD medium containing 0, 1.5, 2.5, 3, 3.5, 4, 4.5, 5, 6, and 6.5 g/L furfural, respectively, and cultured at 30 °C and 170 r/min. The optical density (OD) at 600 nm (OD600) was measured.

The mutant strain *C. tropicalis* YB-3 and the original strain *C. tropicalis* YB-0 were inoculated into the fermentation medium containing 4 g/L, furfural at 6% inoculation amount per generation. It was fermented in a constant temperature shaker at 30 °C and 170 r/min and continuously subcultured for 10 generations. The xylose content in the fermentation broth was determined by HPLC analysis, and the fermentation performance of each generation was compared. Also, the genetic stability of the strain was verified.

### 3.4. Samples Preparation and Transcriptome Analysis

Glycerol-preserved *C. tropicalis* YB-3 was revived on YPD agar (at 30 °C for 48 h to obtain isolated colonies). A single colony was inoculated into 5 mL YPD broth and incubated at 30 °C with 170 r/min agitation for 24 h until the mid-exponential phase. Cultures were divided into two experimental sets. The experimental group was supplemented with 3.0 g/L furfural (final concentration); the control group had no furfural addition. Triplicate biological replicates were maintained per group under controlled conditions (30 °C, 170 r/min). OD600 was tracked hourly from 3 to 5 h post-treatment until both groups reached the mid-log phase. An amount of 1 mL aliquots was collected into pre-chilled 2 mL microtubes and centrifuged at 4 °C, 3500× *g* for 5 min. Pellets were flash-freezed in liquid nitrogen and stored at −80 °C.

Transcriptome analysis of *C. tropicalis* tolerance to furfural was conducted using the reference strain *C. tropicalis* MYA-3404. To minimize biological variation errors, each treated sample had three parallel groups, resulting in six samples from two groups undergoing transcriptome sequencing: control: YB-3P1–P3; treatment: YB-3F1–F3; YB: denotes mutagenesis treatment; 3: the strain screening number; P: blank control group (e.g., P1-P3 represent triplicate controls); F: experimental group (e.g., F1-F3 represent triplicate test samples).

Total RNA was extracted using TRIzol^®^ reagent (TaKaRa Biotechnology Co., Ltd., Dalian, China) following the manufacturer’s protocol, with quality control via NanoDrop ND-1000 (A260/A280 > 1.8, concentration > 50 ng/μL) and Bioanalyzer 2100 (RIN ≥ 7.0), supplemented by agarose gel electrophoresis. Poly(A)+ mRNA was enriched through two rounds of oligo(dT) bead selection, followed by Mg^2+^-mediated fragmentation (94 °C, 6 min) and reverse transcription to cDNA. Double-stranded DNA was synthesized using *E. coli* DNA Polymerase I/RNase H with dUTP incorporation, end-repaired, adenylated, and ligated to Illumina adapters. Libraries were size-selected (300 ± 50 bp) using AMPure XP beads, treated with UDG for strand-specificity, and amplified via 8-cycle PCR (98 °C 15 s, 60 °C 15 s, 72 °C 30 s). Final libraries were sequenced on the Illumina NovaSeq™ 6000 platform (PE150 mode).

For the gene expression analysis, the original data format was fastq, and the fastp software (v0.11.9) was used to control the quality of the original data, including the removal of joints, repetitive sequences, and low-quality sequences. The parameters were the default parameters. The sequencing data were compared to the genome using HISAT2, and the file format was bam. The genes or transcripts were assembled using StringTie software (v2.1.6) and quantified using Fragments Per Kilobase of exon model per Million mapped fragments (FPKM). The differential genes between the samples were analyzed using the R package. The genes with the parameter of false discovery rate (FDR) below 0.05 and absolute fold change ≥ 2 were considered differentially expressed genes. Finally, Gene Ontology (GO) and Kyoto Encyclopedia of Genes and Genomes (KEGG) enrichment analyses of genes were performed using DAVID software (v6.8) [46,47,48,49,50].

### 3.5. RT-qPCR Analysis

For RT-qPCR Primer Design and Normalization, the primers for RT-qPCR were designed using the NCBI Primer-BLAST tool (https://www.ncbi.nlm.nih.gov/tools/primerblast/ (accessed on 3 March 2023)), with their sequences listed in Table S3. The Actin gene (ACTB) served as the endogenous reference gene for data normalization. cDNA was synthesized using the PrimeScriptTM 1st stand cDNA Synthesis Kit (Vazyme Biotech Co., Ltd., Nanjing, China). The amplification system and reaction procedure are as follows: The reaction system was 20 μL, including 10 μL 2×SYBR real-time PCR premixture, 0.4 μL 10 μmol forward and reverse primers, 8.2 μL cDNA template and 1 μL ddH_2_O. The reaction conditions are detailed in Table S4. The relative transcript levels of the target genes were calculated using the 2^−∆∆Ct^ Ct method. Each sample was repeated three times.

### 3.6. Knockout Verification of Furfural Tolerance-Related Genes

Based on the *C. tropicalis* genome and target gene sequences (MET3, GSH, MIPS), homologous arm pairs (upper/lower) were designed using SnapGene (v5.3) (GSL Biotech, Chicago, IL, USA). The GSH gene homologous arms (GSH-T1/GSH-T2), MIPS arms (MIPS-T1/MIPS-T2), and MET3 arms (MET3-T1/MET3-T2) were amplified via PCR using YB-3 genomic DNA as a template with specific primer pairs: GSH: GSH-T1-F1/R1, GSH-T2-F3/R3; MIPS: MIPS-T1-F1/R1, MIPS-T2-F3/R3; MET3: MET3-T1-F1/R1, MET3-T2-F3/R3. The lengths of both the upper and lower homologous arms are approximately 1000 bp. The Kanamycin resistance gene (Kanr) was PCR-amplified from plasmid pET28a using primers: GSH-Kanr-F2/R2, MIPS-Kanr-F2/R2, MET3-Kanr-F2/R2. The lengths of both the upper and lower homologous arms are approximately 900 bp. The primer sequence is shown in Table S5.

Three knockout vectors (pMD20-MET3, pMD20-GSH, pMD20-MIPS) were constructed by inserting respective homologous arm pairs and Kanr cassettes into the linearized pMD20 backbone through Kit C115-01 (Vazyme Biotech Co., Ltd., Nanjing, China) assembly. These vectors were subsequently transformed into strain YB-3 via LiCl-mediated chemical transformation, generating three knockout mutants: Δ*MET3*, Δ*GSH*, and Δ*MIPS*.

Furfural resistance was evaluated by culturing mutants and YB-3 (control) in a medium containing 3.0 g/L furfural. Growth curves were monitored through OD_600_ measurements at 2 h intervals for 24 h under controlled conditions (30 °C, 170 r/min).

### 3.7. Exogenous Verification of Furfural Tolerance Genes MET3, MIPS, and GSH Genes

MET3 and MIPS were cloned into plasmid pCaEXP using Pst I and BamH I (TaKaRa Biotechnology Co., Ltd., Dalian, China) restriction sites. The recombinant plasmids pCaEXP-MET3 and pCaEXP-MIPS were obtained. The GSH gene was cloned into pCaEXP by Pst I and EcoR I (TaKaRa Biotechnology Co., Ltd., Dalian, China) sites. The recombinant plasmid pCaEXP-GSH was obtained. The recombinant plasmids pCaEXP-MET3, pCaEXP-MIPS, and pCaEXP-GSH were transformed into *E. coli* DH5α to obtain *E. coli*-MET3, *E. coli*-MIPS3 and *E. coli*-GSH, respectively. Overnight cultures of wild-type DH5α and recombinant strains were inoculated (1% *v*/*v*) into fresh LB containing 3.0 g/L furfural. The growth curves (OD_600_) were monitored at 1 h intervals for 12 h using a microplate reader (TECAN, Mannedorf, Switzerland) with triplicate biological replicates.

### 3.8. Analytical Methods

All experiments were conducted with three biological replicates. The results were statistically analyzed using SPSS 17.0 (SPSS, Chicago, IL, USA). Data were analyzed using the ANOVA method, and means were significantly different at *p* < 0.05.

## 4. Conclusions

The transcriptomic analysis under furfural stress revealed differentially expressed genes related to metabolic and stress response pathways in *C. tropicalis*, with the MET3, GSH, and MIPS genes being significantly up-regulated. These genes contributed to the maintenance of cell membrane integrity and redox homeostasis, showing a significant positive correlation with the strain’s furfural tolerance. Subsequent experiments could predict tolerance-related genetic elements based on transcriptomic analysis, identify genes and key pathway genes involved in furfural tolerance, and validate them through heterologous expression by knockout or overexpression. It provides a potential target for the transformation and development of more excellent industrial strains with high furfural tolerance to enhance the comprehensive utilization level of biomass resources.

## Data Availability

The original contributions presented in this study are included in the article. Further inquiries can be directed to the corresponding author.

## Supplementary Materials

The following supporting information can be downloaded at: https://www.mdpi.com/article/10.3390/ijms26072999/s1.
