# Supplementary material for: Comprehensive Transcriptomic Analysis of the Isolated Candida tropicalis with Enhanced Tolerance of Furfural Inhibitor
Source: Int J Mol Sci. 2025 Mar 25;26(7):2999. doi: 10.3390/ijms26072999 (PMC11988566; doi:10.3390/ijms26072999)
Supplement: Supplementary file 1 [file ijms-26-02999-s001.zip › ijms-3462429-supplementary.pdf]

Supplementary Material

Table S1 Functional categories and multiples of differentially expressed genes for furfural tolerance in *C. tropicalis* strain YB-3, including transcriptional abundance (in fpkm form)

Gene_id	Description	EC	fc	log2(fc)
CTRG_00614	predicted protein	NA	0.01	-6.15
CTRG_01889	hypothetical protein	EC:1.1.1.283	0.05	-4.32
CTRG_02879	hypothetical protein	EC:3.2.2.23;	0.04	-4.50
CTRG_00519	conserved hypothetical protein	NA	0.03	-4.84
CTRG_00660	hypothetical protein	NA	0.03	-5.29
CTRG_04589	ubiquitin-conjugating enzyme E2-21 kDa 2	EC:2.3.2.23	0.08	-3.69
CTRG_00471	predicted protein	NA	0.01	-6.94
CTRG_01454	hypothetical protein	NA	0.08	-3.71
CTRG_03187	conserved hypothetical protein	NA	0.21	-2.26
CTRG_01809	hypothetical protein	NA	0.01	-7.42
CTRG_05818	predicted protein	EC:2.1.1.59	0.03	-5.19
CTRG_05139	hypothetical protein	EC:1.1.1.358	0.04	-4.74
CTRG_01682	hypothetical protein	EC:3.2.2.3	0.11	-3.18
CTRG_03648	heat shock protein SSA1	NA	0.04	-4.77
CTRG_02980	peroxiredoxin HYR1	EC:1.11.1.24	0.06	-4.05
CTRG_01697	hypothetical protein	NA	0.01	-7.02
CTRG_01656	sulfiredoxin	EC:1.8.98.2	0.01	-7.47

CTRG_01365	conserved hypothetical protein	NA	0.08	-3.74
CTRG_05763	conserved hypothetical protein	NA	0.13	-2.99
CTRG_05773	conserved hypothetical protein	NA	0.09	-3.50
CTRG_02861	hypothetical protein	EC:3.1.1.3	0.06	-4.05
CTRG_02347	hypothetical protein	NA	0.16	-2.66
CTRG_01802	hypothetical protein	EC:3.5.1.52	0.04	-4.62
CTRG_06148	hypothetical protein	EC:2.3.2.23	0.02	-5.93
CTRG_03620	predicted protein	NA	0.00	-7.74
CTRG_02620	hypothetical protein	EC:3.4.21.53	0.08	-3.72
CTRG_01985	conserved hypothetical protein	NA	0.01	-6.87
CTRG_05146	GTPase-activating protein GYP7	NA	0.04	-4.62
CTRG_03485	hypothetical protein	NA	0.03	-5.17
CTRG_00684	conserved hypothetical protein	NA	0.09	-3.46
CTRG_05306	predicted protein	NA	0.16	-2.62
CTRG_01777	predicted protein	NA	0.14	-2.82
CTRG_00788	conserved hypothetical protein	NA	0.03	-4.92
CTRG_04520	conserved hypothetical protein	NA	0.10	-3.31
CTRG_04798	mitochondrial processing peptidase	EC:3.4.24.64	0.08	-3.66
CTRG_01286	conserved hypothetical protein	NA	0.03	-5.12
CTRG_04926	protein precursor	NA	0.11	-3.24
CTRG_03275	hypothetical protein	NA	0.02	-5.77
CTRG_03077	hypothetical protein	NA	0.13	-2.99
CTRG_04975	hypothetical protein	EC:2.1.1.6	0.13	-2.97
CTRG_03474	conserved hypothetical protein	EC:1.3.1.38	0.00	-7.76
CTRG_03841	hypothetical protein	NA	0.15	-2.72

CTRG_01576	conserved hypothetical protein	NA	0.10	-3.31
CTRG_04847	conserved hypothetical protein	NA	0.03	-5.31
CTRG_01817	conserved hypothetical protein	NA	0.01	-6.33
CTRG_05322	hypothetical protein	NA	0.09	-3.40
CTRG_00008	hypothetical protein	NA	0.12	-3.03
CTRG_04067	cell division control protein 48	NA	0.17	-2.52
CTRG_04067	cell division control protein 48	NA	0.17	-2.52
CTRG_03444	predicted protein	NA	0.09	-3.44
CTRG_05182	hypothetical protein	EC:3.4.21.48	0.05	-4.47
CTRG_05506	hydratase-dehydrogenase-epimerase	NA	0.03	-5.19
CTRG_06039	predicted protein	NA	0.02	-5.68
CTRG_02777	conserved hypothetical protein	NA	0.06	-4.03
CTRG_02884	conserved hypothetical protein	NA	0.01	-6.09
CTRG_01730	conserved hypothetical protein	NA	0.03	-5.25
CTRG_04878	predicted protein	NA	0.01	-7.42
CTRG_00080	conserved hypothetical protein	EC:2.3.2.26	0.09	-3.40
CTRG_00610	hypothetical protein	EC:2.5.1.18	0.08	-3.57
CTRG_05774	conserved hypothetical protein	NA	0.06	-4.04
CTRG_05415	hypothetical protein	NA	0.04	-4.82
CTRG_02866	predicted protein	NA	0.09	-3.51
CTRG_01657	conserved hypothetical protein	NA	0.09	-3.51
CTRG_05742	conserved hypothetical protein	NA	0.01	-6.78
CTRG_00587	ribonucleoside-diphosphate	EC:1.17.4.1	0.03	-4.94
CTRG_00015	conserved hypothetical protein	NA	0.07	-3.86
CTRG_05194	conserved hypothetical protein	NA	0.13	-2.95

CTRG_01959	conserved hypothetical protein	NA	0.08	-3.63
CTRG_03840	conserved hypothetical protein	NA	0.06	-4.06
CTRG_05009	DNA repair protein RAD14	NA	0.04	-4.58
CTRG_05716	hypothetical protein	NA	0.03	-5.17
CTRG_03948	conserved hypothetical protein	EC:2.7.11.1	0.02	-5.99
CTRG_02682	glutathione reductase	EC:1.8.1.7	0.13	-2.98
CTRG_00443	predicted protein	NA	0.03	-5.10
CTRG_04324	conserved hypothetical protein	NA	0.08	-3.58
CTRG_05470	conserved hypothetical protein	NA	0.05	-4.31
CTRG_03693	hypothetical protein	EC:2.3.2.31	0.14	-2.84
CTRG_03675	hypothetical protein	EC:3.4.19.12	0.07	-3.85
CTRG_02330	predicted protein	NA	0.00	-7.72
CTRG_05715	ubiquitin-activating enzyme E1 1	EC:6.2.1.45	0.15	-2.72
CTRG_03885	predicted protein	NA	0.01	-7.23
CTRG_01715	conserved hypothetical protein	NA	0.02	-6.01
CTRG_01129	hypothetical protein	NA	0.03	-5.07
CTRG_00508	conserved hypothetical protein	NA	0.07	-3.81
CTRG_05304	conserved hypothetical protein	NA	0.04	-4.49
CTRG_00703	conserved hypothetical	NA	0.06	-4.17
CTRG_05500	hypothetical protein	EC:6.2.1.3	0.01	-7.23
CTRG_00828	conserved hypothetical protein	NA	0.10	-3.39
CTRG_04901	predicted protein	NA	0.03	-4.91
CTRG_01937	inorganic pyrophosphatase	EC:3.6.1.1	6.93	2.79
CTRG_03475	predicted protein	NA	0.04	-4.57
CTRG_02851	predicted protein	NA	0.08	-3.69

Table S2 The transcription levels of the eight genes

Gene	transcript levels
SMS	2.78
AFR1	3.94
ATM1	2.51
GCLM	1.43
G6PD	3.08
GSH	4.31
MET3	13.07
MIPS	8.11

Table S3 qPCR Primer List

Primer Name	Primer Sequence (5'→3')
Actin-F1	GGTGATGGTGTTACCCACGTTGTC
Actin-R1	TCGGTCAAGTCTCTACCAGCCAAG
SMS-F1	TCGGTGGTGGAGATTGTGGA
SMS-R1	GTGATACTTGGCCATTTCCGGT
AFR1-F1	TGGTGTTGTTCCAGGTGGTTT
AFR1-R1	GCTTGAACTCCAACTGCTCCT
ATM1-F1	TGATGAAGCCACATCTGCATTAGA
ATM1-R1	TCGGCATCAGCAATGGTTCG
GCLM-F1	ACGGTGCCAATGGTGCCAATG
GCLM-R1	AGTTTTCCCGTCACATCCTCTTGG
G6PD-F1	TGCCTCCATCTGTTTTCCACACTG
G6PD-R1	AGTTTCCAAATCACGGCCAAATGG
GSH-F1	GCAAGGTTTTGGTGTTGCCA
GSH-R1	CTCTTCTGCGGAAGTTGGGT
MET3-F1	TGAGAGATGAAACTCCTTTGGCT
MET3R1	TGTTCTGGATCACCACGGAA
MIPS-F1	CCGTGCTACATTTTTGGGTGG
MIPS-R1	GGAATTGTTGCGGAGCTGAC

Table S4 Fluorescence quantitative PCR reaction conditions

Step Number	Step Description	Time	Number of Cycles
1	95℃	10 min	1
2	95℃	15 s	
3	58℃	20 s	Go to 2，40×
4	72℃	20 s	
5	Melting Curve Analysis		1

Table S5 Primer sequences used for knockout and verification

Primer Name	Primer Sequence (5'→3')
MET3-T1-F1	ATCTACTAGTCATATGGATTTTAAGATTGCAAAATACGGCAAA
MET3-T1-R1	GCCTCCATGTCCTTGTGATAGTCGTAGTTATAATTAATACCAA
MET3-Kanr-F2	TATCACAAGGACATGGAGGCCCAGAATACC
MET3-Kanr-R2	CGGTACCCGGGGATCCGATTCAGTATAGCGACCAGCATTCACA
MET3-T2-F3	CTGGTCGCTATACTGATGTGTTATTACATAAACTCTCTTATAATTCATAA
MET3-T2-R3	CGGTACCCGGGGATCCGATTATGCCATGAATAAAGATTTGGATCT
GSH -T1-F1	TCTACTAGTCATATGGATATCCGAGTAATGCAGAAGATATACTCC
GSH -T1-R1	AGG
GSH - Kanr-F2	GGGCCTCCATGTCATCAAAATAGAAAAAGCAAAAAAAAAA
	TTTTGATGACATGGAGGCCCAGAATAC
GSH - Kanr-R2	CAGTATAGCGACCAGCATTCACA
GSH -T2-F3	GAATGCTGGTCGCTATACTGTAATTTACTCATACTTGATTATATTAAATAGAAAAAA
GSH -T2-R3	GGTACCCGGGGATCCGATATCAACAAAAAGGAAATACCCGTTTTATT
MIPS-T1-F1	TCTACTAGTCATATGGATATCAACTAGTTATGGGTAAACTAAATCCTTGC
MIPS -T1-R1	CCTCCATGTCGGTATGAAGATTGAATACTAGTTATAGAAAGG
MIPS - Kanr-F2	TCTTCATACCGACATGGAGGCCCAGAATACC
MIPS - Kanr-R2	CTATCAGTATAGCGACCAGCATTCACA
MIPS -T2-F3	GCTGGTCGCTATACTGATAGAGAATCAAACAAATAATAAATAATAATTTTG
MIPS -T2-R3	GGTACCCGGGGATCCGATATCCCCACAACTTGTCTATTCATATGATG
MET3 -F1	ATGCCTATTCCAGCTCCACACG
MET3 -R1	CTAGTAGAAACCTTGATCTTTCAAATAACTG
MIPS -F1	ATGTCTATTAACTTCAATTCTACTAAAGCTCA
MIPS -R1	TTACTGAGTAACTTCTTGCAATCTCAAG
GSH -F1	ATGTTTAATAGACAATTCTTGACAAAATCG
GSH -R1	TTATGTCATGGTGACCAACTCTTCTG

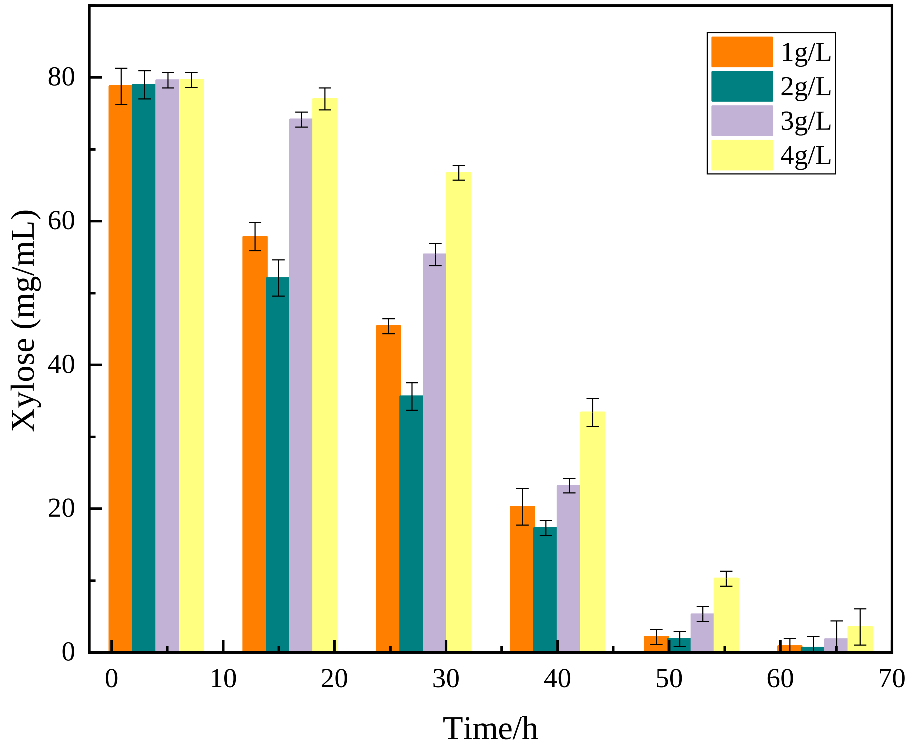

Figure S1 Comparison of fermentation performances of xylose consumption by mutant strains *C. tropicalis* YB-3 at different furfural concentrations

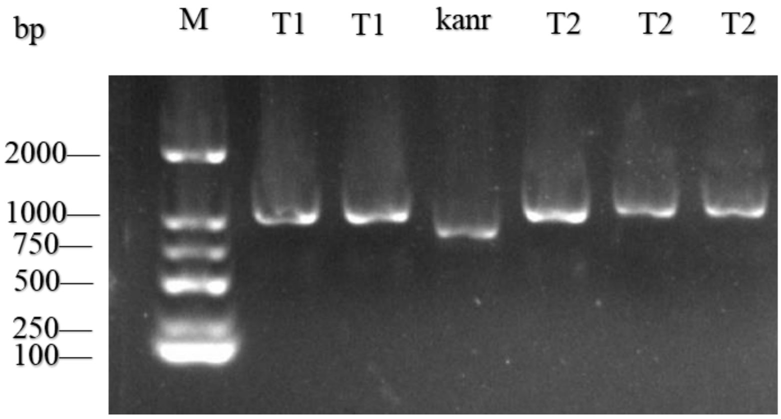

(a) GSH-T1, GSH-T2 and GSH-Kanr

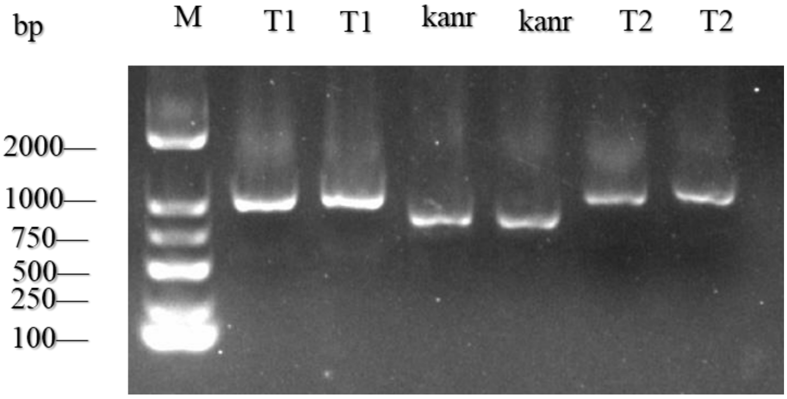

(b) MIPS-T1, MIPS-T2 and MIPS–Kanr

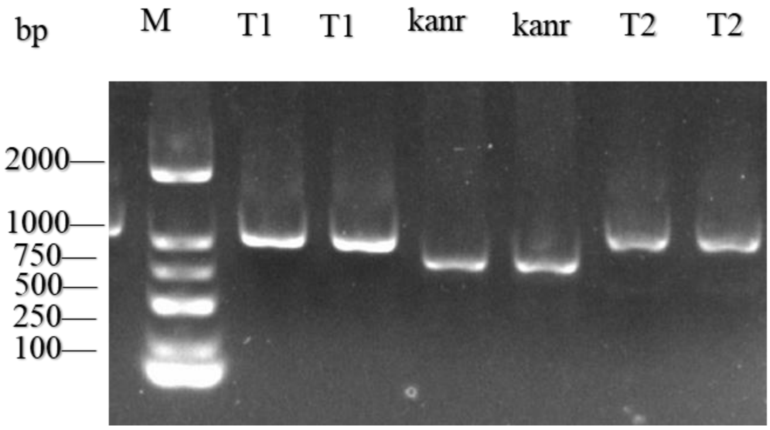

(c) MET3-T1, MET3-T2and MET3-Kanr

Figure S2 PCR validation of upper and lower homologous arms and resistance genes. The homologous arm pairs GSH-T1/GSH-T2 (for the GSH gene), MIPS-T1/MIPS-T2

(for the MIPS gene), and MET3-T1/MET3-T2 (for the MET3 gene) were amplified via PCR using *C. tropicalis* genomic DNA as the template. Agarose gel electrophoresis revealed distinct bands of approximately 1000 bp (Fig.S2(a), (b), (c)), which closely matched the theoretical size of the target fragments. Subsequently, the Kanamycin resistance gene (Kanr) was amplified from the plasmid pET28a template using primer pairs GSH-Kanr-F2/R2, MIPS-Kanr-F2/R2, and MET3-Kanr-F2/R2. A distinct band of 900 bp was observed (Fig.S2(a), (b), (c)) corresponding to the expected size of the Kanr coding sequence

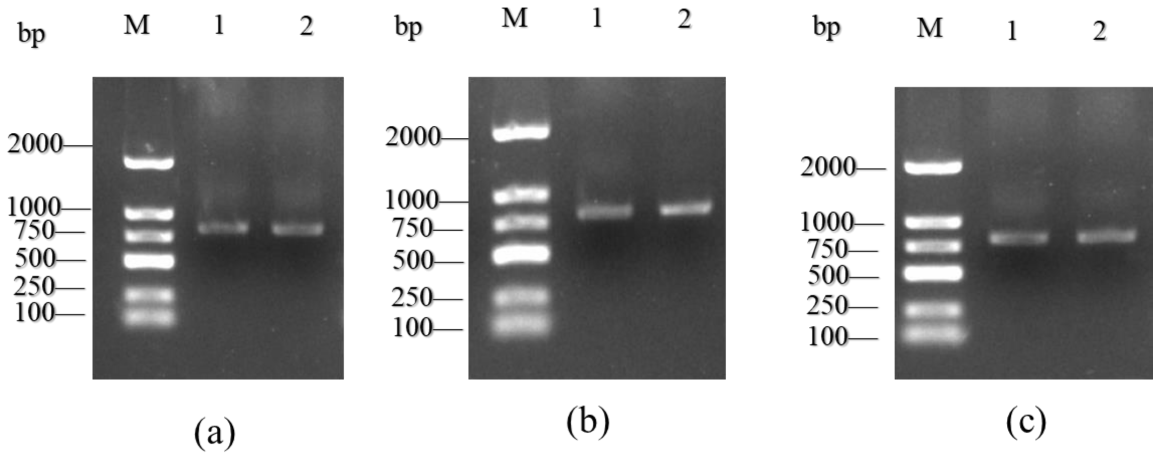

Figure S3 Knockout carrier identification. Lane M: DNA Marker; Lanes 1-2: Amplification of the Δ*MET3* knockout strain using primers MET3-Kanr-F2 and MET3-Kanr-R2, yielding a 900 bp band (Kanr1). (b) Lane M: DNA Marker; Lanes 1-2: Amplification of the Δ*GSH* knockout strain using primers GSH-Kanr-F2 and GSH-Kanr-R2, yielding a 900 bp band (Kanr2). (c) Lane M: DNA Marker; Lanes 1-2: Amplification of the Δ*MIPS* knockout strain using primers MIPS-Kanr-F2 and MIPS-Kanr-R2, yielding a 900 bp band (Kanr2)
